# Hemoglobin-Dilution Method: Effect of Measurement Errors on Vascular Volume Estimation

**DOI:** 10.1155/2017/3420590

**Published:** 2017-10-02

**Authors:** Matthew B. Wolf

**Affiliations:** Department of Pharmacology, Physiology and Neuroscience, University of South Carolina School of Medicine, Columbia, SC 29209, USA

## Abstract

The hemoglobin-dilution method (HDM) has been used to estimate changes in vascular volumes in patients because direct measurements with radioisotopes are time-consuming and not practical in many facilities. The HDM requires an assumption of initial blood volume, repeated measurements of plasma hemoglobin concentration, and the calculation of the ratio of hemoglobin measurements. The statistics of these ratio distributions resulting from measurement error are ill-defined even when the errors are normally distributed. This study uses a “Monte Carlo” approach to determine the distribution of these errors. The finding was that these errors could be closely approximated with a log-normal distribution that can be parameterized by a geometric mean (*X*) and a dispersion factor (*S*). When the ratio of successive Hb concentrations is used to estimate blood volume, normally distributed hemoglobin measurement errors tend to produce exponentially higher values of *X* and *S* as the SD of the measurement error increases. The longer tail of the distribution to the right could produce much greater overestimations than would be expected from the SD values of the measurement error; however, it was found that averaging duplicate and triplicate hemoglobin measurements on a blood sample greatly improved the accuracy.

## 1. Introduction

Vascular volume changes have been estimated from blood hemoglobin concentration ([Hb]_*b*_) changes for some time. Dill and Costill [1] in 1974 used this method along with hematocrit changes to estimate the changes in the volumes of blood (BV), plasma (PV), and red cells (RCV) due to dehydration. In 1987, Hahn [2] used a somewhat different approach of using changes in [Hb]_*b*_ to estimate BV variation during transurethral prostatic surgery, where both fluid uptake into and blood loss from the vascular system occurred; he called this approach the “hemoglobin-dilution method” or HDM. Modification of HDM has been used to calculate allowable blood loss under hemodilution conditions [3], to estimate BV changes during hemodialysis [4], to determine the PV kinetic effects of isotonic and hypertonic PV expanders [5], and to detect hypovolemia and dehydration in patients by using volume loading [6] and numerous other applications.

HDM and its modifications all require calculation of the ratio of [Hb]_*b*_ values at various time points compared to an initial [Hb]_*b*_ determination; however, the effect of measurement errors on the accuracy of these volume determinations has never been adequately explored. This task is not as easy as it might appear as the statistical distribution of these ratios is not straightforward. The simple assumption that the measurement errors are normally distributed does not lead to a statistically normal distribution, but to a “Cauchy,” “ratio,” or “Lorentzian” distribution, which cannot be parameterized like a normal distribution since it contains infinite values when the denominator goes to zero [7]. The purpose of the present study to us is a “Monte Carlo” approach to investigate the statistical properties of the ratio of [Hb]_*b*_ values when used in a volume determination and show how the errors can be much larger than expected.

## 2. Materials and Methods

As an illustration of the potential errors using the [Hb]_*b*_-ratio approach in volume determinations, consider the estimate of vascular volumes prior to fluid therapy in a patient in a critical-care setting. The gold standard for initial BV estimation is to use radioisotopes for this determination, but this procedure is rarely done because of the cost, patient safety, and the time required.

### 2.1. Blood Volume (BV)

The proposed approach to BV estimation requires (1) initial (*i*) measurement of blood hemoglobin concentration ([Hb]_*b*_^*i*^), (2) a measured volume (*V*_inf_) of a non-Hb-containing solution infused over a period of several minutes, and (3) a final (*f*) measurement ([Hb]_*b*_^*f*^). Assuming that neither Hb nor the volume of infused solution is lost from the circulation over the relatively short time between measurements and that the blood is well mixed before the final measurement, conservation of Hb yields

(1) $$\begin{array}{c} {\left[\;Hb\;\right]}_{b}^{i}\times {BV}^{i}={\left[\;Hb\;\right]}_{b}^{f}\times \left(\;{BV}^{f}+V_{inf}\;\right) \end{array}$$

which can be solved for BV^*i*^ as

(2) $$\begin{array}{c} {BV}^{i}=V_{inf}\times \frac{1}{{\left[Hb\right]}_{b}^{i}/{\left[Hb\right]}_{b}^{f}-1}. \end{array}$$

An inherent assumption in this method and other methods using measurements of [Hb]_*b*_ or hematocrit at various time points is that the *F*_cell_ factor (ratio of large vessel to total-body hematocrit) remains constant between initial and final [Hb]_*b*_ measurements.

As seen, a measurement of the [Hb]_*b*_ ratio in the denominator of (2) is required for the determination. The larger this ratio, the less the effect of measurement errors. For example, if error-free BV^*i*^ = 5 l and *V*_inf_ = 1 l, then the error-free [Hb]_*b*_ ratio in (2) would be 1.2 (20% dilution). If *V*_inf_ = 0.5 l, the ratio would be only 1.1 (10% dilution). A 1%  [Hb]_*b*_-ratio overestimation in the first case would result in a ratio of 1.212, hence, a volume estimation of 4.72 l (5.6% error), whereas the same error in the second case would result in an estimate of only 4.5 l (10% error). The same kind of reasoning applies when using the [Hb]_*b*_ ratio to determine the common approach of sequential changes in blood or plasma volume [5, 6], but the non-Hb-containing fluid lost from the circulation does not affect the accuracy of this kind of estimation.

### 2.2. Plasma Volume (PV)

If the infused solution does not contain any protein and does not leave the plasma (*p*), and negligible protein is lost from plasma over the short time between measurements, then conservation of protein (Pr) requires

(3) $$\begin{array}{c} {\left[\;Pr\;\right]}_{p}^{i}\times {PV}^{i}={\left[\;Pr\;\right]}_{p}^{f}\times \left(\;{PV}^{f}+V_{inf}\;\right). \end{array}$$

Equation (3) can be solved for PV^*i*^ as

(4) $$\begin{array}{c} {PV}^{i}=V_{inf}\times \frac{1}{{\left[Pr\right]}_{p}^{i}/{\left[Pr\right]}_{p}^{f}-1}. \end{array}$$

If error-free PV^*i*^ = 3 l (Hct = 40%) and *V*_inf_ = 1 l, then the error-free concentration ratio in (4) would be 1.33. If *V*_inf_ = 0.5 l, the ratio would be 1.17. Again, measurement errors would more dramatically affect the estimation accuracy in the latter case; however, equivalent measurement errors would affect BV^*i*^ estimations to a greater extent (see above). Alternatively, albumin concentrations could be used in (4).

### 2.3. Red-Cell Volume (RCV)

(5) $$\begin{array}{c} {RCV}^{i}={BV}^{i}-{PV}^{i}. \end{array}$$

Clearly, the accuracy of RCV^*i*^ estimation would be affected by estimation accuracy of both BV^*i*^ and PV^*i*^. Hence, RCV^*i*^ estimation is most affected by measurement errors.

### 2.4. Infusion Solutions

An infusion solution that meets the requirements for all three volume estimations is 6% Hetastarch in 0.9% saline. McIlroy and Kharasch [8] infused 1 l of this solution over a 7-8 min period into healthy males after a 900 ml blood draw. Using the HDM, they found that over the 20 min period after the infusion, the estimated BV increase stayed at about 1 l. Hence, in the present study, an infusion interval as long as 10–20 min would satisfy the requirements of the approach. Morgan [9] lists a number of other possible nonprotein, colloid solutions and many new ones have been marketed recently.

### 2.5. Statistical Determination of Volume-Estimation Errors

First, assuming that BV^*i*^ and [Hb]_*b*_^*i*^ have error-free baseline values of 5 l and 150 g/l, respectively, and that the value of *V*_inf_ is 1 l (*V*_inf_/BV^*i*^ = 0.2), then from (2), [Hb]_*b*_^*f*^ would be 125 g/l. To assess the potential effects of measurement-precision errors, an independent, normally distributed, random value was added to each of these two [Hb]_*b*_ measurements in (2) and then (2) was solved for the resulting BV^*i*^ value. This procedure was repeated 10,000 times on an Excel spreadsheet (available upon request). The code for generating these random numbers in Excel was *A∗*NORMINV(RAND()(0,1)). This statement produces a number picked from a normal distribution with a mean of one and an SD equal to the value of *A*. From the 10,000 estimated values of BV^*i*^, a histogram was generated in Excel using bin sizes of 0.2 l. These data were transferred to the SigmaPlot computer program (SigmaPlot, Systat Software, San Jose, CA) where they were plotted and fit by mathematical equations.

In this study, the statistical-distributional measures for initial BV, PV, and RCV were determined for variations in (1) the measurement-precision error, (2) changes in the *V*_inf_/BV ratio, (3) the effect of averaging multiple [Hb]_*b*_ measurements on a blood sample, and (4) decreased hematocrit values often seen in critical-care patients.

## 3. Results

### 3.1. Measurement-Precision Errors

**Figure 1:**
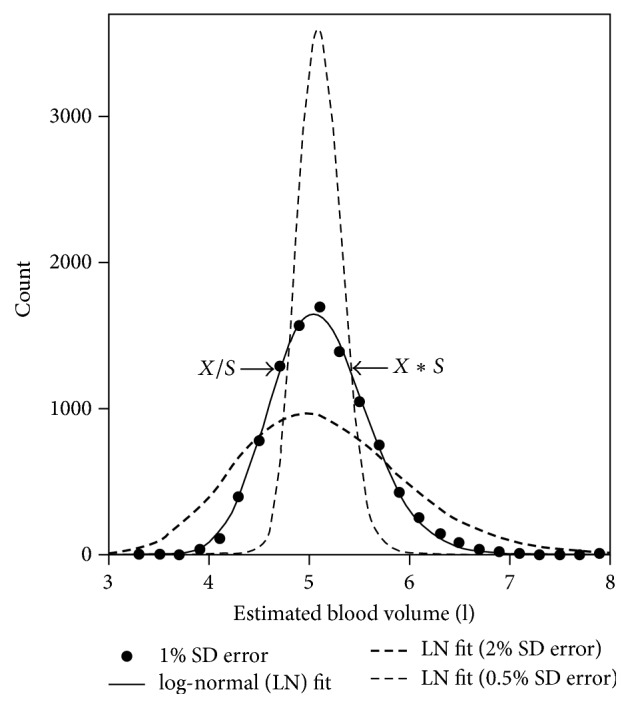
A histogram of computed initial blood volume (BV) estimates assuming an error-free BV of 5 L for normally distributed [Hb]_*b*_ measurement errors of 0.5, 1, and 2% SD is shown. The solid circles are histogram counts of the number of error occurrences (10,000 total) which fall in the 0.2 l wide bins for the 1% SD added error as computed with an Excel spreadsheet. The solid line is a log-normal (LN) fit to these data. The thin- and thick-dashed lines are fits to the 0.5 and 2% added error data (not shown), respectively. The arrows indicate the bounds for 68.3% of the potential errors (5 − *X*/*S* to 5 + *X∗S*) for the 1% SD case, where *X* is the geometric mean value and *S* is the dispersion factor (see text) for the LN distribution.

Histogram data (solid circles) of the distribution of the estimated 10,000 BV^*i*^ values are plotted in Figure 1 for a [Hb]_*b*_ measurement-precision error of 1% SD. These data are closely fit by a log-normal (LN) distribution as shown by the solid line, even though, theoretically, this distribution is not LN [7]. Hence, even though the added error is normally distributed, the distributions are not Gaussian (normal). Also in Figure 1, LN fits to histogram data for errors of 0.5% SD (thin-dashed line) and 2% SD (thick-dashed line) are shown. The correlation coefficients for these fits were all > 0.995. As the error increases, the shape of the distribution becomes more asymmetrical with the peak moving to the left and becoming flatter and a longer tail forming on the right.

The LN distribution can be characterized [10] as having a log-mean (*μ*) and log-SD (*σ*) determined as for a normal distribution, except that instead of using the individual data, *x*, log ⁡(*x*) is used. If the natural log is selected, then *μ* and *σ* can be back-transformed into the geometric mean (*X*) and the multiplicative dispersion factor (*S*) by taking each of these quantities to the power of the exponential function (*e*). Hence, *X* = *e*^*μ*^ and *S* = *e*^*σ*^. For normally distributed measurement errors, 68.3% of the possible observations lie between the values, mean ± SD. In contrast, for a LN distribution, the SD equivalence is *X*/*S* to *X∗S*. The arrows in Figure 1 point to these BV^*i*^ boundaries. These measures were used to characterize all the distributions determined in the present study.

Statistics for the distributions of BV, PV, and RCV for 0.5, 1, and 1.5% SD errors are given in Table 1 for error-free measurements of *X* = 5 l and *S* = 1. As seen, these errors only slightly increase the *X* value of these distributions from the error-free value. For example, an increase in the measurement error from 0.5% to 1.5% SD produced a shift in the estimated *X* value for BV from 5 l to only 5.05 l. In contrast, *S* increased much more, from 1.04 to 1.14. The result is that the error-bounds range goes from 8.4% to 26%, a 3-fold increase. The effect on PV estimation is less as seen in Table 1. As expected, RCV estimation is most affected, producing a small decrease in *X*, but a quite large increase in *S* from 1.12 to 1.47; the error-bounds range increased from 23 to 79%, suggesting that the accuracy of RCV estimation by this method is compromised unless measurement errors are minimized. It is apparent from the data in Table 1 that the % errors in these estimations are skewed towards overestimation of volumes.

### 3.2. Effects due to Decreasing Relative Infusion Volume (*V*_inf_/BV)

As described above, estimation errors increase as the *V*_inf_/BV ratio decreases. The effects of decreasing this quantity are shown in Table 2. As seen, decreasing this ratio down to as low as 70% of its standard value of 1 l led to generally small increases in the estimation errors for all three volumes. As expected, the RCV estimation had the greatest increase.

### 3.3. Effects of Multiple Measurements

The first three lines of Table 3 show the effects of averaging multiple measurements on a blood sample. It is clear that this approach is an effective way to decrease estimation errors for all volumes, but particularly those for RCV. As seen, the errors decreased approximately as the square-root of the number of averaged sample values.

### 3.4. Effects of Lowered Hematocrit, [Hb]_*b*_, and [Pr]_*p*_

It is expected that that the estimation errors could increase by lowering any of the three critical quantities, hematocrit, [Hb]_*b*_, or [Pr]_*p*_. To look at the effects of the worst case of decreasing all three quantities, estimation errors were assessed for an initial hematocrit of 30%, [Hb]_*b*_ = 120 g/l, and [Pr]_*p*_ = 50 g/l. The bottom line of Table 3 shows the effect when triplicate measurements are made. As seen, this averaging effect limited the errors to only slight increases, even with these large changes in critical quantities.

## 4. Discussion

The aim of this study was to show the possible errors when using the [Hb]_*b*_-concentration ratio to determine vascular volumes. The example used was the estimation of pretherapy volume in a patient in order to specify appropriate fluid therapy. The same kinds of errors would occur in studies determining sequential changes in blood or plasma volume [5, 6]. A major finding in the present study was that adding normally distributed, independent, random error to [Hb]_*b*_ measurements produced a distribution of BV estimates which were closely fit by a log-normal (LN) curve. The shape of this distribution (Figure 1) became more asymmetric as the measurement-precision error increased; the peak flattened and a long tail formed to the right (higher values). This effect is characterized by an increased dispersion factor (*S*) as shown in Table 1. The dispersion errors also increased in PV estimation (Table 1) but were less than those for BV because the volume infused was a greater percentage of PV. The dispersion errors were the greatest for RCV estimation because they were influenced by both BV and PV estimation errors. The latter errors coupled with the smaller volume of RCV compared to the other volumes produced the greatest potential % errors of the three volumes (Table 1).

Another important factor that increases errors is the relative infusion volume (*V*_inf_/BV) as shown in Table 2. Inability to infuse sufficient volume is one cause and possible loss of the infused fluid from the circulation during the rapid infusion is a second cause. It could be that some patients cannot tolerate infusions equivalent to 20% of their blood volume. Although a 1 l infusion of 6% hetastarch was well tolerated in normal subjects [8], this volume may not be tolerated in some ill patients, but using a lesser volume would result in increased estimation errors. Likewise, it is possible that the hetastarch or some other colloid may leave the circulation more rapidly during the infusion in severely ill patients, resulting in potentially increased estimation errors.

Critically ill patients often have low hematocrit and hemoglobin concentration values along with low concentrations of plasma proteins; however, the finding (see Table 3) was that although these factors increased the volume-estimation errors, the increases were not overly large. The most important way to minimize volume-estimation errors in any study using concentration ratios is to average multiple laboratory measurements of [Hb]_*b*_ and [Pr]_*p*_. This observation is clearly shown in Table 3. Only practicality limits the number of these repeated measurements.

The volume-estimation errors examined in this study were caused by the worst kind of measurement error, independent and uncorrelated. Measurement errors due to zero drift or change in sensitivity of the measuring instrument would likely be correlated; that is, measurement error of initial and final measurements would be in the same direction. The result would be to diminish the tails of the LN distribution and hence the dispersion (*S*) values. It is possible to simulate such effects, but that was not done in the present study.

A proposed approach to determining the statistics of measurement-precision errors is for each laboratory to make a number of measurements on a given sample. The result is that each laboratory could establish and possibly minimize their potential volume-estimation errors.

## 5. Conclusions

The error analysis done in this study is applicable to any technique where measurements are made of ratios of hemoglobin, plasma protein concentration, or hematocrit in order to determine changes in fluid volumes (see (2) and (4)). As shown in the present study, normally distributed measurement errors lead to potential volume-estimation errors far greater than the SD of the measurement errors. Such error analyses have not been done for the numerous studies estimating vascular volume changes using this approach.

## Figures and Tables

**Table 1:** Statistical measures of log-normal (LN) distributions for estimation of initial volumes of blood (BV), plasma (PV), and red cells (RCV) due to measurement-precision errors.

Added- error SD (%)	BV (l) ^†^*X* /*∗* *S*	^‡^% BV error /*∗* *S*	PV (l) *X* /*∗* *S*	% PV error /*∗* *S*	RCV (l) *X* /*∗* *S*	% RCV error /*∗* *S*
0.5	5 /*∗* 1.04	−4.1 to +4.3	3 /*∗* 1.03	−2.8 to +2.9	2 /*∗* 1.12	−11 to +12
1	5.02 /*∗* 1.09	−8.4 to +8.8	3.01 /*∗* 1.06	−5.5 to +5.9	1.97 /*∗* 1.27	−21 to +27
1.5	5.05 /*∗* 1.14	−12.0 to +14.0	3.01 /*∗* 1.09	−8.2 to +8.9	1.94 /*∗* 1.47	−32 to +47

Error-free BV = 5 l and PV = 3 l (Hct = 40%); [Hb]_*b*_ = 150 g/l, [Pr]_*p*_ = 70 g/l; *V*_inf_/BV = 0.2; ^†^*X* is the geometric mean and *S* is the dispersion (shape) factor of the volume distributions; /*∗* signifies the bounding values of 68.3% of the volume values that are in the range of *X*/*S* to *X∗S*; ^‡^the range of % errors for /*∗*  *S* bounds.

**Table 2:** Statistical measures of LN distributions for estimation of initial BV, PV, and RCV when the relative infusion volume (*V*_inf_/BV) is decreased.

*V*_inf_/BV	BV (l) *X* /*∗* *S*	% error */∗* *S*	PV (l) *X* /*∗* *S*	% error */∗* *S*	RCV (l) *X* /*∗* *S*	% error */∗* *S*
0.2	5.02 /*∗* 1.09	−8.4 to +8.8	3.01 /*∗* 1.06	−5.5 to +5.9	1.97 /*∗* 1.27	−21 to +27
0.17	5.02 /*∗* 1.11	−9.4 to +10	3.01 /*∗* 1.06	−6.2 to +6.6	1.97 /*∗* 1.32	−24 to +32
0.14	5.02 /*∗* 1.12	−11.0 to +12.0	3.01 /*∗* 1.08	−7.2 to +7.7	1.94 /*∗* 1.4	−29 to +40

1% SD error added to [Hb]_*b*_ and [Pr]_*p*_ measurements; see Table 1 for other error-free conditions and description of statistical quantities.

**Table 3:** Statistical measures of LN distributions for initial volume estimations when multiple measurements are made on a sample.

Measurement (s)	BV (l) *X /∗* *S*	% error */∗* *S*	PV (l) *X /∗* *S*	% error */∗* *S*	RCV (l) *X /∗* *S*	% error */∗* *S*
Single	5.02 /*∗* 1.09	−8.4 to +8.8	3.01 /*∗* 1.06	−5.5 to +5.9	1.97 /*∗* 1.27	−21 to +27
Duplicate	5 /*∗* 1.06	−5.8 to +6.2	3 /*∗* 1.04	−4.0 to +4.1	1.98 /*∗* 1.18	−15 to +18
Triplicate	5 /*∗* 1.05	−4.7 to +5.0	3 /*∗* 1.03	−3.2 to +3.3	1.99 /*∗* 1.14	−13 to +14
^†^Triplicate	5 /*∗* 1.05	−4.8 to +5.0	3.5 /*∗* 1.05	−3.6 to +3.7	1.48 /*∗* 1.21	−17 to +21

1% SD error added to [Hb]_*b*_ and [Tpr] measurements; *V*_inf_/BV = 0.2; ^†^Hct = 30%, [Hb]_*b*_ = 120 g/l, [Pr]_*p*_ = 50 g/l; error-free PV and RCV = 3.5 and 1.5 l, respectively; see Table 1 for other error-free conditions and description of statistical quantities.
